# National prevalence of IC/BPS in women and men utilizing veterans health administration data

**DOI:** 10.3389/fpain.2022.925834

**Published:** 2022-08-24

**Authors:** Jennifer T. Anger, Kai B. Dallas, Catherine Bresee, Amanda M. De Hoedt, Kamil E. Barbour, Katherine J. Hoggatt, Marc T. Goodman, Jayoung Kim, Stephen J. Freedland

**Affiliations:** ^1^Department of Urology, UC San Diego Health, San Diego, CA, United States; ^2^City of Hope Urology, Duarte, CA, United States; ^3^Department of Biostatistics and Bioinformatics Research Center, Cedars-Sinai Medical Center, Los Angeles, CA, United States; ^4^Urology Section, Department of Surgery, Veterans Affairs Medical Centers Division of Population Health, Durham, NC, United States; ^5^National Center for Chronic Disease Prevention and Health Promotion, Centers for Disease Control and Prevention, Atlanta, GA, United States; ^6^Center for Study of Healthcare Implementation, Innovation and Policy Veterans Health Administration, Los Angeles, CA, United States; ^7^Cedars-Sinai Comprehensive Cancer Institute, Cedars-Sinai Medical Center, Los Angeles, CA, United States; ^8^Department of Surgery and Biomedical Science, Cedars-Sinai Medical Center, Los Angeles, CA, United States

**Keywords:** interstitial cystitis, bladder pain syndrome, IC/BPS, epidemiology, prevalence, sex/gender

## Abstract

**Importance:**

Interstitial cystitis/bladder pain syndrome (IC/BPS) is an immense burden to both patients and the American healthcare system; it is notoriously difficult to diagnose. Prevalence estimates vary widely (150-fold range in women and >500-fold range in men).

**Objectives:**

We aimed to create accurate national IC/BPS prevalence estimates by employing a novel methodology combining a national population-based dataset with individual chart abstraction.

**Study design:**

In this epidemiological survey, all living patients, with ≥2 clinic visits from 2016 to 2018 in the Veterans Health Administration, with an ICD-9/10 code for IC/BPS (*n* = 9,503) or similar conditions that may represent undiagnosed IC/BPS (*n* = 124,331), were identified (other were controls *n* = 5,069,695). A detailed chart review of random gender-balanced samples confirmed the true presence of IC/PBS, which were then age- and gender-matched to the general US population.

**Results:**

Of the 5,203,529 patients identified, IC/BPS was confirmed in 541 of 1,647 sampled charts with an IC/BPS ICD code, 10 of 382 charts with an ICD-like code, and 3 of 916 controls. After age- and gender-matching to the general US population, this translated to national prevalence estimates of 0.87% (95% CI: 0.32, 1.42), with female and male prevalence of 1.08% (95% CI: 0.03, 2.13) and 0.66% (95% CI: 0.44, 0.87), respectively.

**Conclusions:**

We estimate the prevalence of IC/BPS to be 0.87%, which is lower than prior estimates based on survey data, but higher than prior estimates based on administrative data. These potentially represent the most accurate estimates to date, given the broader and more heterogeneous population studied and our novel methodology of combining in-depth chart abstraction with administrative data.

## Introduction

Interstitial cystitis/bladder pain syndrome (IC/BPS) is defined as an unpleasant sensation perceived to be related to the urinary bladder persisting for more than 6 weeks, in the absence of infection or other identifiable causes (1, 2). The burden of IC/BPS on the American public is immense in both human and financial terms (1, 2).

Many diagnostic tests for IC exist and include urinalysis, urine culture, potassium sensitivity testing, cystoscopy, a biopsy of the bladder wall, and hydrodistention of the bladder (3).

However, none of these tests can definitively diagnose IC. Thus, differentiating IC from other conditions is still a challenge, and objective markers are urgently needed to definitively diagnose the condition. A major diagnostic hallmark of IC is the absence of identifiable causes for symptoms; for example, a urinary tract infection (UTI). Yet, many patients have a sudden presentation of IC that mimics a bladder infection. In addition, the diagnosis of IC is often delayed because patients present nonspecific symptoms that may reflect unrelated disorders. It can take up to 4–5 years from the first visit to definitively diagnose IC (4–7). Another feature complicating the diagnosis of IC is the fact that patients with IC often have a disease “flare”, or exacerbations of symptoms that are unpredictable and debilitating. There is great variability between patients about flare length, frequency, and severity (8).

Due to the lack of definitive diagnostic criteria or tests for IC/BPS, as well as symptomatic overlap with other conditions (e.g., overactive bladder), the true prevalence of IC/BPS is difficult to determine (3, 9, 10). Prevalence estimates of IC/BPS fluctuate widely based on the study methodology and diagnostic criteria used (5). In general, estimates are lower in studies based on physician diagnosis and higher in studies utilizing survey data (5). A prior administrative claims study in a managed care population found a prevalence of 0.045 to 0.197% in women and 0.008 to 0.041% in men (11) depending on the IC definition used. In contrast, a population-based telephone study found from 2.7% (based on “high sensitivity” definition) to 6.5% (based on “high specificity” definition) of women and 4.2% (based on “high sensitivity” definition) to 1.9% (based on “high specificity” definition) of men may have IC (12, 13).

Thus, contemporary IC/BPS prevalence estimates in the current literature varies by a nearly 150-fold range in women (0.045–6.5%) and a >500-fold range in men (0.008–4.2%) (1, 5, 11, 12, 14).

The lack of accuracy in estimating the national prevalence of IC/BPS is a major limitation to progress in this field. The overall aim of this study was to determine an accurate national prevalence estimate of IC/BPS by employing a novel methodology that overcomes the limitations of prior studies.

## Methods

We sought to develop a nationally representative longitudinal cohort of subjects with IC. To accomplish this, we took full advantage of the largest national integrated health care system in the country: the Veterans Health Administration (VA) (www.va.gov/health). The VA provides healthcare to 8,920,000 veterans, has >1,700 sites of care, and employs >300,000 full-time staff. While each site only has access to patients seen at that site, all data from all sites are collated in the VA Informatics and Computing Infrastructure. VINCI contains the entire medical record for each patient, including but not limited to all diagnoses (ICD-9 and ICD-10 codes), all billing codes, all medications prescribed, as well as date filled, all laboratory tests, all radiology reports, all pathology reports, all vitals, including height, weight, and blood pressure measurements, home address, demographics, and all clinic/procedures/surgery notes. By including data containing all ICD-9 diagnoses and all billing codes on nearly 9 million veterans, VINCI has the strength of a very large administrative database. However, containing detailed medical records (i.e., clinic notes), VINCI has the strengths of an individual-level chart review. We sought to capture all IC diagnoses within the entire VA system. We then reviewed the individual charts in depth to confirm the diagnosis of IC. Using the subset that underwent detailed chart review and the group of confirmed patients with IC, we then extrapolated these to the entire VA system and from there to the entire country. Thus, using the unique strengths of the VA integrated medical network, we can accurately estimate the national prevalence of IC for men and women.

After obtaining IRB approval (number: Pro00041326), VINCI was used to identify all living patients with at least two clinic visits at least 6 weeks apart from 2016 to 2018 with ICD-9/10 codes for IC/BPS (595.1/ N30.10, N30.1, N30.11) (*n* = 9,503). To identify similar conditions that may represent undiagnosed or misdiagnosed IC/BPS, we also created a separate “other pelvic pain” cohort that included diagnosis codes for including prostatitis (601.9, N41.9 men only), vaginismus (625.1, N94.2), vulvar vestibulitis (625.71, N94.810), vulvodynia (625.7, N94.819 women only), and dyspareunia (525.0, N04.1, men and women) (*n* = 124,331). All other patients were considered controls (*n* = 5,069,695).

Using a random number generator, we selected a subset of these patients to review in depth (random gender-balanced sample). We aimed to perform an in-depth chart review (the patient's complete medical record in the VA system) in at least 1,500 patients with a code for IC/BPS or “other pelvic pain” conditions based on our prior pilot study (11). We ensured that at least 80% (*n* = 1,200) came from the pool of subjects with an actual ICD-9/10 code of IC/BPS (i.e., assessing for overdiagnosis) and 20% (*n* = 300) came from the pool of subjects with ICD-9/10 code for an IC/BPS- “like”/other pelvic pain condition. The goal of 1,500 subjects was based on a power calculation that provided 80% of power to construct a 95% confidence interval (CI) of the estimated prevalence in this enriched subset at +/−0.05 or less, assuming (conservatively) at least 50% of this enriched subset of subjects is positive for IC/BPS.

Our criteria for a correct/actual diagnosis of IC/BPS were if at least one of the following criteria was met:

1. Two visits (in the VA system) complaining of unpleasant bladder-centric sensation in the absence of positive urine culture at least 6 weeks apart. This is based on the American Urological Association's definition of IC/BPS as bladder-centric pain that lasts at least 6 weeks in duration.
2. One visit complaining of bladder-centric pain/unpleasant bladder-centric sensation and a second visit complaining of “likely” IC/BPS-related bladder symptoms in the absence of positive urine culture at least 6 weeks apart (both at the VA). We defined “likely” IC/BPS-related pain as pain that could be due to IC/BPS but without a specific complaint of bladder-centric pain or bladder tenderness on exam. Symptoms of “likely” IC/BPS include dysuria, pelvic pain, chronic lower abdominal pain, dyspareunia, urinary frequency, or urgency.
3. A history of bladder pain and/or a history of IC/BPS (in which the visit was outside the VA system) with one additional VA visit, complaining of bladder-centric pain in the absence of a positive urine culture.
4. Based on sampling weights, the prevalence was estimated for the VA population. Adjustments were performed as there are known baseline demographic differences between the patient population with VHA and the general population (15). Further gender and age adjustments were calculated to estimate the US prevalence based on the 2010 Census data (16).

In cases where it was impossible to definitively determine if the patient truly had IC/BPS by our criteria (e.g., patient had a diagnosis code for IC /BPS but there was a lack of information or ambiguity in the medical record), the cases were classified as “equivocal”. Rather than exclude this group (and potentially underestimate prevalence), a sensitivity analysis was performed by repeating our prevalence calculations with the assumption that the patients who were equivocal did have IC/BPS. The analysis was performed using SAS Enterprise Guide v7.1 software.

## Results

**Figure 1:**
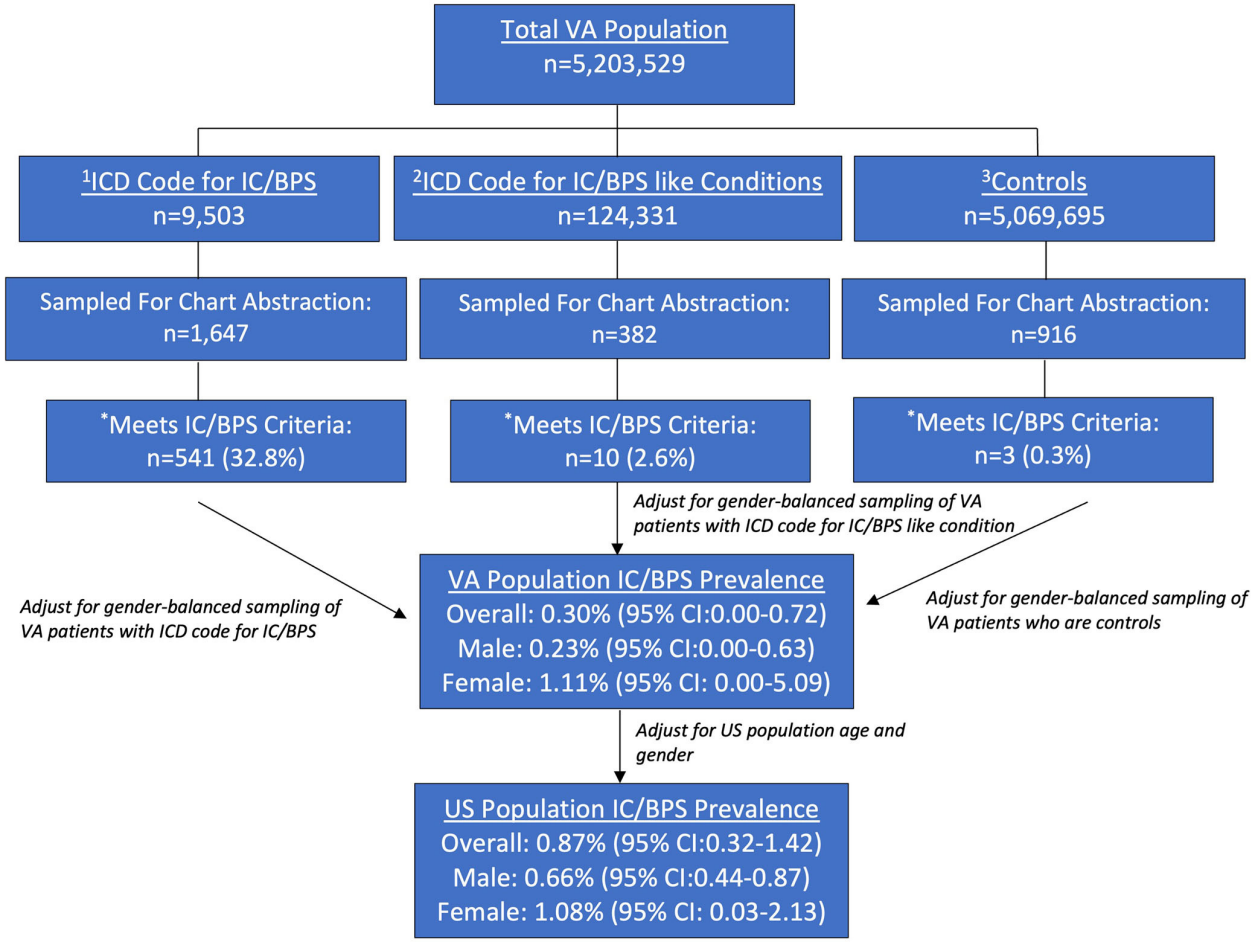
Estimated prevalence of IC/BPS in the VHA and US Population. ^1^ICD-9 and ICD-10 diagnosis of IC/BPS (595.1/N30.10). ^2^IC/BPS- “like” conditions which include prostatitis dyspareunia, vaginismus vulvodynia, and vulvar vestibulitis. ^3^Defined as all patients not meeting criteria 1 and 2. *Criteria for diagnosis of IC/BPS.

Of the 5,203,529 identified patients with active VHA, 9,503 had a code for IC/BPS, 124,331 had a code for another pelvic pain condition, and 5,069,695 had neither. Of the 1,647 sampled charts with a code for IC/BPS that underwent thorough chart review, 541 (32.8%) met diagnostic criteria for IC/BPS (736 or 44.7% including equivocal cases). Of the sample of 382 patients with a code for an “IC/BPS-like” condition that underwent a thorough chart review, 10 (2.6%) met the criteria for IC/BPS (11 or 2.9% including an equivocal case). Of the 916 sampled controls, 3 (0.3%) met the criteria for IC/BPS (there were no equivocal controls, Figure 1) after a thorough chart review.

After adjustment for the size of the sampling pools using gender and age, the overall prevalence of IC/BPS in the VHA population was estimated to be.30% (95% CI: 0.00, 0.72) with the female and male prevalence of 1.11% (95% CI: 0.00, 5.09) and.23% (95% CI: 0.00, 0.63), respectively. After adjustment for age and gender-matching to the US general population, these values translated to national prevalence estimates of.87% (95% CI: 0.32, 1.42), with female and male prevalence of 1.08% (95% CI: 0.03, 2.13) and.66% (95% CI: 0.44, 0.87), respectively (Figure 1).

Within the VHA, women had a similar prevalence of IC/BPS by age strata (1.11% overall and 1.62% for <40 years, 0.89% for 40–49 years, 1.43% for 50–59 years, 0.36% for 60–60 years and.13 for >70 years). This is in contrast to men, where the majority of patients with IC/BPS were <40 years old. While men <40 years had a prevalence of 1.64%, all other strata had IC/BPS rates of 0.02–0.07% (Table 1).

**Table 1:** VHA and national prevalence of IC/BPS.

	**Strict Criteria**		**Equivocal Criteria**	
**Prevalence**	Rate*	**95% CI**	**Rate**	**95% CI**
Overall**	0.30	(0.00, 0.72)	0.33	(0.00, 0.82)
*Male*	0.23	(0.00, 0.63)	0.24	(0.00, 0.73)
<40yr	1.64	(0.00, 0.63)	1.64	(0.00, 0.73)
40–49yr	0.03	(0.00, 2.86)	0.03	(0.00, 2.87)
50–59yr	0.02	(0.00, 0.22)	0.03	(0.00, 0.29)
60–69yr	0.07	(0.00, 0.18)	0.08	(0.00, 0.28)
70+	0.05	(0.00, 0.53)	0.08	(0.00, 0.60)
*Female*	1.11	(0.00, 5.09)	1.23	(0.00, 6.06)
<40yr	1.62	(0.00, 7.04)	1.69	(0.00, 7.62)
40–49yr	0.89	(0.00, 6.23)	1.08	(0.00, 7.67)
50–59yr	1.43	(0.00, 4.66)	1.59	(0.00, 5.63)
60–69yr	0.36	(0.00, 2.94)	0.48	(0.00, 3.92)
70+	0.13	(0.00, 1.12)	0.22	(0.00, 1.87)

VHA; Veterans Health Affairs, IC/BPS; interstitial cystitis/bladder pain syndrome.

*Rate per 100 persons.

**By strict criteria, overall US Standardized rate was 0.87 (95% CI: 0.32,1.42) overall, 0.66 (95% CI: 0.44, 0.87) for men and 1.08 (95% CI: 0.03, 2.13) for women. By Equivocal criteria, overall US Standardized rate was 0.94 (95% CI: 0.32,1.56) overall, 0.67 (95% CI: 0.58, 0.75) for men and 1.20 (95% CI: 0.00, 2.39) for women.

Our sensitivity analysis revealed similar estimates of VHA and national IC/BPS estimates when applying strict vs. strict plus equivocal criteria for IC/BPS diagnosis (Table 1).

## Discussion

In this study, we estimated the national prevalence of IC/BPS by a novel methodology combining analysis of a large population-based dataset with an in-depth chart review. We estimate the national prevalence of IC/BPS to be 0.87% (1.08 and 0.66% for women and men, respectively). We believe our findings represent a more accurate estimate of the true prevalence of IC/BPS than prior reports as our approach addresses many of the known challenges in studying the epidemiology of IC/BPS. This is both due to the broader and more heterogeneous population we studied, as well as our use of a novel approach to confirming administrative population-based data with individual chart reviews.

Given our methodology, it is not surprising that our IC/BPS prevalence estimates are higher than those reported in prior purely administrative studies but lower than those relying on survey data. For example, Clemens et al., in a managed care population, estimated a prevalence ranging from 0.045 to 0.197% for women and between 0.008 and 0.041% for men (11). On the other hand, estimates of IC/BPS prevalence in US women aged ≥18 from the RAND IC Epidemiology Study (RICE), which was based on surveys from >100,000 US households, were 2.7% (high specificity) and 6.5% (high sensitivity) for women and 1.9% (high specificity) and 4.2% (high sensitivity) for men (5, 12, 14). Similarly, prevalence rates of IC/BPS from the Boston Area Community Health (BACH) survey of 5,506 men and women were estimated to be 1.3% in men and 2.6% in women (17).

While most prior studies estimated the prevalence of IC/BPS to have a female-to-male ratio of at least 5:1, we report a more balanced gender ratio (1.6:1). This is similar to the findings in the RICE and BACH surveys, where the male-to-female ratio of IC/BPS prevalence was approximately 1.5:1 and 2:1, respectively, (12, 14, 17). Prevalence of IC/BPS varied by age strata and gender. Women aged <40–59, years had a slightly higher prevalence than other groups, while IC/BPS was most prevalent in men <40 years of age. In contrast to our findings, both RICE and BACH reported a similar prevalence in men by age stratum (12, 14, 17). A possible explanation for this is the potential impact of Post Traumatic Stress Disorder or other factors resulting in a higher prevalence of IC/BPS among male veterans (16). By survey, the prevalence in South Korea appears to be lower than in the US at 0.26%. A survey of providers in Japan also found the prevalence of IC/BPS to be low, and also found a female-to-male ratio of 1:5.8. Survey data in Finland was found to be an order of magnitude higher (450/100,000). These studies outside the US identify significant variation in prevalence, which may be due to race, geography, awareness of the disease, and, when clinical data are used, diagnostic patterns (18–20). Further work using methods similar to ours is needed to validate our findings in other countries and healthcare systems.

Despite its strengths, there are limitations to this study. First, it is not known if results from the VHA population can be extrapolated to the general population. However, given that the VHA database consists of a large, heterogenous population, including women, we believe our results are robust. We are also limited to the data and documentation available in patients' records. Fortunately, the impact of this limitation is likely minimal as our sensitivity analysis of IC/BPS equivocal cases did not produce appreciably different prevalence estimates.

In conclusion, we present what we believe to be the largest and most comprehensive effort to accurately estimate the national prevalence of IC/BPS to date. Our strategy of combining administrative claims data analysis with in-depth chart abstraction overcomes many limitations of prior studies.

A. Two visits (in the VA system) complaining of unpleasant bladder-centric sensation in the absence of positive urine culture at least 6 weeks apart.
B. One visit complaining of bladder-centric pain/unpleasant bladder-centric sensation and a second visit complaining of “likely” IC/BPS-related pain in the absence of positive urine culture at least 6 weeks apart (both at the VA). We defined “likely” IC/PBS-related pain as pain that could not be due to IC/BPS but without a specific complaint of bladder-centric pain or bladder tenderness on exam. Symptoms of “likely” IC/BPS include dysuria, pelvic pain, chronic lower abdominal pain, and dyspareunia.
C. A history of bladder pain and/or a prior diagnosis of IC/BPS (outside the VA system) with one additional visit complaining of bladder-centric pain in the absence of a positive urine culture.
D. Patients were considered negative for IC/BPS when there was a history of urologic/gynecologic cancer to avoid overestimating IC/BPS prevalence, as it is possible that pain was due to cancer.

## Data availability statement

The raw data supporting the conclusions of this article will be made available by the authors, without undue reservation.

## Ethics statement

The studies involving human participants were reviewed and approved by Cedars-Sinai IRB Durham VA IRB. Written informed consent for participation was not required for this study in accordance with the national legislation and the institutional requirements.

## Author contributions

JA, JK, SF, MG, and KH contributed to conception and design of the study. CB and AD organized the database. CB and KD performed the statistical analysis. KD, JA, JK, and SF drafted the manuscript. MG, KH, AD, and KB wrote sections of the manuscript. All authors contributed to the article and approved the submitted version.

## Funding

This work was funded by the Center of Disease Control and Prevention, Grant Number U01DK111226.

## Conflict of interest

The authors declare that the research was conducted in the absence of any commercial or financial relationships that could be construed as a potential conflict of interest.

## Publisher's note

All claims expressed in this article are solely those of the authors and do not necessarily represent those of their affiliated organizations, or those of the publisher, the editors and the reviewers. Any product that may be evaluated in this article, or claim that may be made by its manufacturer, is not guaranteed or endorsed by the publisher.

## Author disclaimer

The findings and conclusions in this report are those of the authors and do not necessarily represent the official position of the Centers for Disease Control and Prevention.
